# The Cerebellar Gene Database: a Collective Database of Genes Critical for Cerebellar Development

**DOI:** 10.1007/s12311-022-01445-w

**Published:** 2022-07-20

**Authors:** Miguel Ramirez, Joshua Wu, Matthew Liu, Derek Wu, Dave Weeden, Daniel Goldowitz

**Affiliations:** 1 Centre for Molecular Medicine and Therapeutics, BC Children’s Hospital Research Institute, 950 W 28th Ave, Vancouver, BC V6H 3V5 Canada; 2 University of British Columbia, Vancouver, BC V6T 1Z4 Canada

**Keywords:** Cerebellum, Genes, Database, Disorders, Mouse, Human

## Abstract

This report presents the first comprehensive database that specifically compiles genes critical for cerebellar development and function. The Cerebellar Gene Database details genes that, when perturbed in mouse models, result in a cerebellar phenotype according to available data from both Mouse Genome Informatics and PubMed, as well as references to the corresponding studies for further examination. This database also offers a compilation of human genetic disorders with a cerebellar phenotype and their associated gene information from the Online Mendelian Inheritance in Man (OMIM) database. By comparing and contrasting the mouse and human datasets, we observe that only a small proportion of human mutant genes with a cerebellar phenotype have been studied in mouse knockout models. Given the highly conserved nature between mouse and human genomes, this surprising finding highlights how mouse genetic models can be more frequently employed to elucidate human disease etiology. On the other hand, many mouse genes identified in the present study that are known to lead to a cerebellar phenotype when perturbed have not yet been found to be pathogenic in the cerebellum of humans. This database furthers our understanding of human cerebellar disorders with yet-to-be-identified genetic causes. It is our hope that this gene database will serve as an invaluable tool for gathering background information, generating hypotheses, and facilitating translational research endeavors. Moreover, we encourage continual inputs from the research community in making this compilation a living database, one that remains up-to-date with the advances in cerebellar research.

**Supplementary Information:**

The online version contains supplementary material available at 10.1007/s12311-022-01445-w.

## Introduction

The cerebellum is one of a few structures in the mammalian brain possessing a discrete set of neurons that can be assigned to specific functions relative to their neurotransmitter phenotype (i.e., glutamatergic or GABAergic) and has a focused output on motor control. Thus, perturbations of these highly ordered arrays can be easily assessed at both anatomical and functional levels. These qualities of the cerebellum made it one of the first tractable regions of the brain to identify (and then screen) for mutations that resulted in aberrant morphology and motor function. The first concerted cataloging of the genetic loci that impact cerebellar function was part of a larger pioneering effort that culminated in 1965 with the publication of the “Catalog of the Neurological Mutant of the Mouse” by Richard Sidman, Margaret Green, and Stanley Appel [1]. This catalog led to a relative flood of research papers in the 1970s and beyond, largely in France, Japan, and the USA, that detailed the use of mice that had mutations in these loci to elucidate the genetic bases of cerebellar development and function. Around the time of the Mouse Mutant Catalog [1], there began a pioneering effort to catalog the mutations in humans that resulted in aberrant function. Victor McKusick published the first catalog of Mendelian Inheritance in Man in 1966 [2]. Since then, a large number of Mendelian mutations in humans affecting cerebellar structure and function have been logged into the modern version of the human catalog — “Online Mendelian Inheritance in Man” (OMIM, https://omim.org/). With the arrival of gene cloning and sequencing technologies that culminated in the full sequences of the human and mouse genomes, the genes responsible for these mutants have been identified.

Resources for the examination of gene expression at anatomical and molecular levels over time have provided valuable tools to probe genes of interest [3–7]. The recent spate of single-cell/nuclei RNAseq data over developmental time has greatly enriched this knowledge environment [8–12]. We and others provided early efforts to identify genes altered in single-gene mutations that impacted cerebellar development [5, 13–16]. These advances in gene databases are matched with the exciting advances in our understanding of cerebellar function, particularly in the realm of non-motor function [17–24].

With this publication, we sought to cull these datasets to highlight the many genes in the mouse and human that are critical to cerebellar development. Importantly, we sought to see how the work in the mouse intersects with efforts to understand genes involved in human cerebellar development and functions. We see this as an initial and ever-expandable dataset to be used as a reference to explore the genetic bases of cerebellar development and function in mice and humans.

## Methods

### Mouse Genome Informatics (MGI) Database Mining

We used the “Genes and Markers Query” tool (http://www.informatics.jax.org/marker) for our search for genes with cerebellar phenotypes in mice using the following parameters: Using the “Mouse phenotypes and mouse models of human disease” search function, we used the term “cerebellum.” This search returned a list of 549 genes with potentially a cerebellar phenotype. Phenotypes were then retrieved for cerebellar genes by using the “MGI Batch Query” tool on the MGI website. For the output of this search, we chose “Nomenclature” under Gene Attributes, and “Mammalian Phenotype (MP)” under Additional Information. The input for this analysis was the 549 genes identified in the initial query. The references/sources for phenotypes were retrieved from “Mammalian Phenotype Ontology Annotations” for each gene. To filter these phenotypes for those found within the cerebellum, we constructed a list of cerebellar and brain centric terms to use as parameters (Table 1). For phenotypes that were not specific to the cerebellum, the referenced study was examined to identify whether the phenotype was specifically observed in the cerebellum. If the phenotype was found in the cerebellum, it was kept. A gene was removed from the database if none of the phenotypes occurred in the cerebellum.

**Table 1:** Cerebellar- and brain-centric terms used to filter phenotypes retrieved from MGI. Terms in this table served as an initial filter for cerebellar phenotypes. If a phenotype contained one of these terms, it was considered for further examination

MGI phenotype filters			
Cerebellum	Interneuron	Axon	Coordination
Cerebellar	Golgi cell	Dendrite	Balance
Cerebellar granule cell	Basket cell	Synapse	Spatial
Purkinje cell	Stellate cell	Synaptic	Movement
Rhombic lip	Glia	Neurotransmitter	Gait
Neuroepithelium	Unipolar brush cell	Neuron specification	Learning
Neuronal precursor	Neuron differentiation	Reflex	Memory

### PubMed Literature Search

For a subset of mouse genes, we identified cerebellar phenotypes through a literature search in PubMed (https://pubmed.ncbi.nlm.nih.gov/). Articles were individually curated for studies that have previously created a knockout mouse for a given gene and identified a cerebellar phenotype. This literature search has been conducted over the years by the lab and has previously been used as a database of references for cerebellar genes. In total, 161 PubMed articles were accessed to identify genes that when perturbed result in a cerebellar phenotype. In the second section of the database, “Mouse Cerebellar Phenotypes,” genes/phenotypes with a listed PubMed ID (PMID) in the “Source” column are genes that were identified in our PubMed literature search but were not recorded in MGI as having a cerebellar phenotype. Genes/phenotypes that overlapped between the two were given the corresponding MGI Phenotype ID and J number for their phenotypes and sources, respectively.

### OMIM Database Mining

To identify loci associated with human disorders with cerebellar phenotypes, we conducted a comprehensive search on the Online Mendelian Inheritance in Man (OMIM) database (https://www.omim.org/). We utilized the primary search function, using “cerebellum” as the keyword. From the result of this search, we downloaded the “Gene Map Table” containing “Phenotype Only Entries.” A table containing human disorders with cerebellum included in their phenotypes and their associated loci was retrieved from this search. To refine the list of human disorders to those with a cerebellar phenotype, the term “cerebellum” was entered into the “Clinical Synopsis Advanced Search” tool on OMIM (https://www.omim.org/search/advanced/clinicalSynopsis). We used this list of disorders to filter our Gene Map Table for phenotypes with a cerebellar phenotype by matching OMIM Phenotype ID numbers.

## Results

### Database Access and Summary

**Fig. 1:**
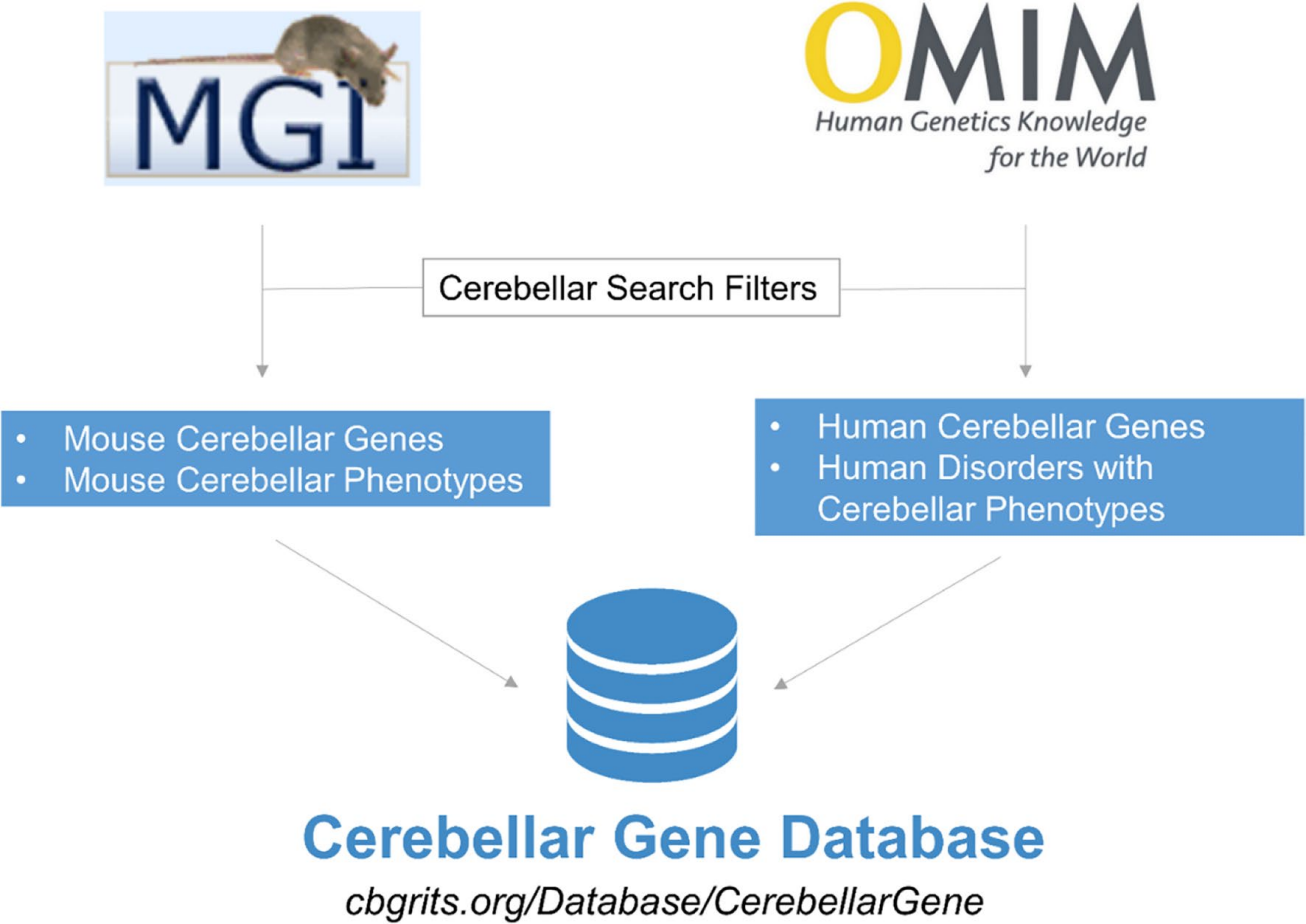
Flow chart describing the input sources and data extracted from these databases during the construction of the Cerebellar Gene Database. The bottom displays the URL that can be used to access the database online

The Cerebellar Gene Database can be accessed online at https://cbgrits.org/Database/CerebellarGene. A summary of the input sources and how they contributed to the Cerebellar Gene Database is outlined in Fig. 1. The provided “User Guide” (Supplementary File 1) details how to navigate the online resource and outlines the various capabilities such as filtering, exporting, and contributing.

### Constructing a Mouse Cerebellar Gene Database from MGI and PubMed

In this section of the database, we sought to amass an atlas of genes that, when knocked out in the mouse, resulted in a cerebellar phenotype. Our first step was to mine the Mouse Genome Informatics (MGI) database (http://www.informatics.jax.org/), an international database resource for the laboratory mouse which provides integrated genetic, genomic, and biological data. We conducted an initial search for protein coding genes that may have a cerebellar phenotype which resulted in an initial list of 549 genes. The phenotypes as a result of perturbation of these genes were then filtered for those found within the cerebellum. To do this, we constructed a list of cerebellar- and brain-centric terms to use as parameters (Table 1). Filtering resulted in a list of 504 genes with a potential cerebellar phenotype.

We then retrieved the references for each of these phenotypes from MGI. These were hand-annotated for phenotypes that did not specifically involve the cerebellum. For example, the term “abnormal axon morphology” (MP:0005404) may have been highlighted as a cerebellar phenotype when in fact it may not have necessarily occurred in the cerebellum but only as part of the overall pleiotropic outcome of the mutant gene. After this filtering step, our list consisted of **464** genes and **213** unique ontologically defined phenotypes retrieved from the MGI database. To compile a comprehensive list of cerebellar genes and their related phenotypes, a set of genes that were identified directly from research articles in PubMed were added. During the construction of this database, the list of genes identified by our literature search stood at 150 genes from 126 articles. We found that several genes in our PubMed-derived list overlapped with the MGI cerebellar database, identifying **94** genes that were not recorded in MGI with a cerebellar phenotype. We then combined the genes identified through MGI with our literature-based curated list and obtained a total of **543** genes and **213** unique phenotypes.

General information about these mouse genes is provided in the first section of the online Cerebellar Gene Database entitled “Mouse Cerebellar Genes.” For each gene, the data table provides the gene name, chromosomal location, starting and ending positions, centimorgan (cM) location, strand, and the MGI ID with a link to the dedicated webpage on MGI (Table 2).

**Table 2:** Overview of the contents of the “Mouse Cerebellar Genes” section of the Cerebellar Gene Database

Column name	Description
Gene symbol	Gene symbol as appears on MGI
Gene name	Name of the gene as appears on MGI
Chromosome	Chromosome in which the gene is located
Start	Starting position of the gene in the corresponding chromosome
End	Ending position of the gene in the corresponding chromosome
cM	Gene in centimorgans
Strand (GRCm38)	Strand orientation of the gene (exists as “ + ” or “ − ”)
MGI ID	Unique ID number given to each gene by MGI (http://www.informatics.jax.org/marker/MGI)

The retrieved cerebellar phenotypes and their corresponding genes are detailed in the second section of the online Cerebellar Gene Database entitled “Mouse Cerebellar Phenotypes.” This table includes the associated gene name, MGI Gene ID and MGI Phenotype ID to a corresponding webpage on MGI, and the source as a MGI J number (Table 3).

**Table 3:** Overview of the contents of the “Mouse Cerebellar Phenotypes” section of the Cerebellar Gene Database

Column name	Description
Gene symbol	Gene symbol (as appears on MGI)
Gene name	Name of the gene (as appears on MGI)
MGI ID	Unique ID number given to each gene by MGI
Phenotype	Phenotype implicated in gene’s loss of function (e.g., knockout), unless specified otherwise
Mouse phenotype	Unique ID number given to each phenotype by MGI
Source	ID number (from MGI or PubMed) of study/publication detailing the phenotype observed in the gene’s loss of function

### Identifying a Comprehensive Catalog of Cerebellar Human Disorders and Associated Loci

We then compiled a catalog of human disorders with cerebellar phenotypes and associated genomic loci. To do so, we searched the Online Mendelian Inheritance in Man (OMIM) database (https://omim.org/) to identify any disorders with a cerebellar phenotype described in the clinical synopsis. Additionally, we retrieved information on any loci that have been previously associated with these disorders. We identified **682** human disorders involving a cerebellar phenotype associated with **630** mutant loci that result in a cerebellar phenotype in humans. As validation for our approach, we identified several well-known human disorders with cerebellar phenotypes such as Joubert syndrome, Dandy-Walker syndrome, and spinocerebellar and Friedreich ataxias [25]. We also opted to include disorders with evidence of pleiotropy, in which the cerebellar abnormalities were one of several phenotypes.

The third section of the Cerebellar Gene Database, entitled “Human Cerebellar Genes,” details information about the genes/loci associated with human disorders with cerebellar phenotypes including the gene/locus symbol and name, cytogenetic location, genomic coordinates, OMIM IDs with a corresponding link to the gene webpage on OMIM, comments from OMIM about mutations related to the corresponding phenotype, and the mouse ortholog (Table 4).

**Table 4:** Overview of the contents of the “Human Cerebellar Phenotypes” section of the Cerebellar Gene Database

Column name	Description
Gene symbol/locus	Gene or locus symbol as appears on OMIM
Gene/locus name	Name of the gene and/or locus associated with the phenotype
Gene/locus MIM number	ID used to search the gene/locus directly on OMIM
Cytogenetic location	Cytogenetic location of the gene associated with the phenotype
Genomic coordinates (NCBI/GRCh38)	Location of the gene, denoted in the format of [Chromosome]:[Starting position]-[Ending position]
Disorder/phenotype	Name of disorder or phenotype as can be found on OMIM
Phenotype MIM number	ID number of the disorder/phenotype on OMIM
Inheritance	Inheritance pattern of the disease/phenotype
Phenotype Map Key	A number from 1–4 stands for the following: “(1) the disorder was positioned by mapping of the wild-type gene; (2) the disease phenotype itself was mapped; (3) the molecular basis of the disorder is known; (4) the disorder is a chromosome deletion or duplication syndrome,” retrieved from https://www.omim.org/help/faq
Comments	Comments/additional information regarding the phenotype
Mouse gene (from MGI)	Mouse homolog (as can be found on MGI) of the gene responsible for the human phenotype

Ortholog inferences were retrieved from MGI, which uses orthologs defined by the Alliance of Genome Resources using the DRSC Integrative Ortholog Prediction Tool (DIOPT). DIOPT integrates human, mouse, fly, worm, zebrafish, and yeast ortholog predictions made by 12 different algorithms based on sequence homology, phylogenetic trees, and functional similarity. DIOPT ranks orthologs based on a score indicating the number of tools that support a given orthologous gene-pair relationship. MGI defines an ortholog using the DIOPT stringent set of criteria: “An ortholog called by 3 or more methods is included if it is a best count or a best reverse count.” This section of the database also includes information on the human disorders with cerebellar phenotypes including inheritance pattern, OMIM IDs with links to OMIM webpages describing the disorder, and a Phenotype Map Key. The Phenotype Map Key from OMIM indicates a score of 1–4 corresponding to the following: (1) the disorder was positioned by mapping of the wild-type gene; (2) the disorder itself was mapped; (3) the molecular basis of the disorder is known; or (4) the disorder is a chromosome deletion or duplication syndrome.

### Connecting Mouse Models and Human Cerebellar Genes and Disorders

Identifying genes that, when perturbed in mice and humans, result in a cerebellar phenotype allowed us to examine the proportion of human genes that have been investigated using the mouse as a model system. We identified that **603** of the disorder-associated human loci involved a mutation in a single gene. Among these genes, **588** (97.5%) have an ortholog in the mouse, indicating that many of these genes are highly conserved. Strikingly, when compared to the cerebellar mouse gene list, only **126/588** (20.9%) of these genes have previously been knocked out in the mouse and have an observed cerebellar phenotype. This unexpected finding indicates that the majority of conserved genes that result in a cerebellar abnormality in humans have yet to be studied in the context of the mouse cerebellum. We predict that investigating these genes in the mouse model system will provide insights into the etiology of the cerebellar phenotypes observed in these disorders. The genes overlapping between mouse and human cerebellar genes represent only 27.2% (**126**/**464)** of the cerebellar mouse genes that have a human ortholog associated with a human disorder with a cerebellar phenotype. The remaining 74.8% of the cerebellar mouse genes may result in perturbed cerebellar function in humans; however, variants within these genes have yet to be identified. Supplementary Table 1 summarizes the genomic location, mouse phenotypes, human disorders, and general classification and functions (Gene Ontology) of the 126 overlapping genes. Expectedly, these genes are enriched for GO terms “cerebellum development” and “cerebellum morphogenesis,” serving as validation for our approach. This analysis has revealed that overlapping genes are enriched for functions such as calcium ion transport, neurite growth, and myelination (Supplemental Fig. 1). In Supplementary Table 1, we also provide links to online sources describing expression pattern in mice from the Gene Expression Database (http://www.informatics.jax.org/expression.shtml) and in humans from the Protein Expression Atlas (https://www.proteinatlas.org/). Overall, our database will serve as a rich resource of potential candidate genes for sequencing for cerebellar disorders/phenotypes with unknown genetic origin and for investigation in the mouse model for their potential role in cerebellar development and function.

## Discussion

The Cerebellar Gene Database is a timely and novel contribution to the cerebellar research community. Our curated database is the first resource, to our knowledge, that consists solely of genes critical for cerebellar development and function; and catalogs their phenotypes when perturbed in both mouse and humans. Larger gene databases, such as MGI and OMIM, are typically more comprehensive and lack focus on a particular tissue or subregion of a given tissue. In the context of the cerebellum, Sato et al. [6] previously developed the Cerebellar Development Transcriptome Database (CDT-DB). While this database provides a useful tool for evaluating temporal and spatial expression patterns in the cerebellum and provides links to several other resources, this database does not curate genes specifically important for the cerebellum and lacks any phenotypic information. The Cerebellar Gene Database thus provides a novel and central resource that has been missing in the field.

In comparing mouse and human cerebellar genes, we identified a vast disparity between the gene lists. The result of this comparison provides a list of genes to be studied in the mouse model or candidates for human disorders with cerebellar phenotypes without a known genetic origin. However, it is important to acknowledge that this discrepancy may be partially due to cerebellar defects being missed for a variety of reasons. The researchers may not be experts in cerebellar phenotypes or simply were focused on other regions of the brain. Many phenotypes and their associated genes may not have been documented in MGI or OMIM. Phenotypes may also have been overlooked in previous studies due when motor-deficits were not obvious. More recently, the cerebellum has been associated with non-motor functions which highlights the importance evaluating/re-evaluating the impact of cerebellar phenotypes cognitive functions [22, 26]. The Cerebellar Gene Database will undoubtedly help facilitate these efforts, driving forward our understanding of cerebellar function.

With the aspiration that the study of mouse models of genetic disorders can be translated into the development of diagnostic and/or therapeutic interventions, it is important to understand the etiology of the genetic disorders in question. Examples abound of genes initially discovered in mice that are later shown to inform human etiology, as well as the usefulness of mouse models in further elucidating the mechanisms by which genes initially discovered in humans drive disease processes.

An example of how findings in the mouse lead to discoveries in the human is *Zic1* which was first studied in mice due to its exclusive expression in the nervous tissue. Within the cerebellum, *Zic1* is abundantly expressed in the granule cells [27]. Subsequent studies revealed that *Zic1* is involved in the neurogenesis of granule cells. Significantly, heterozygous deletion of the homologous genes *ZIC1* and *ZIC4* has been shown to be involved in the Dandy-Walker malformation [13], a neurodevelopmental disorder characterized by hypoplasia of the cerebellar vermis and cystic dilation of the fourth ventricle. The discovery of the importance of *Zic1*/*ZIC1* in cerebellar development exemplifies the use and importance of mutant mouse models in illuminating the etiology of human diseases.

On the other hand, there are also examples of genes first identified in humans that are then explored in the mouse. Of particular note is the exploration of the cerebellum’s role in the etiology of ASD. A prime example is the tuberous sclerosis complex (TSC). Mutations in the human *TSC1* and *TSC2* genes are often attended with comorbid phenotypes associated with autism spectrum disorders (ASDs) [28]. These initial findings in the human presented exciting potential for exploration in mouse models as an approach to discover the syndromic causes of ASD and possible therapeutic strategies. Through the generation of mutant mouse strains harboring conditional knockout of either the *Tsc1* or *Tsc2* gene in the cerebellar Purkinje cells (PCs) [29, 30], the causative roles of the *TSC* genes in ASDs have been demonstrated with promising outcomes. Namely, the loss of either *Tsc1* or *Tsc2* in the cerebellar PCs results in ASD-like phenotypes including repetitive behaviors and severe social behavior deficits. But importantly, the investigators discovered that administration of the *mTOR* inhibitor, Rapamycin, can prevent many of the ASD-like phenotypes exhibited by the mutant mice. These seminal studies illustrate the effectiveness of mutant mouse models in not only uncovering disease mechanisms, but also identifying potential therapeutic strategies. Additionally, the ability to use conditional knockouts involving the targeted elimination of a specific gene in a specific cell type (in this case, the PCs) highlights the ability for a finer dissection of genetic causation using mouse models and identifies a solid link between cerebellar perturbations and the etiology as well as the pathology of ASDs.

Our hope in amalgamating the Cerebellar Gene Database is to inform and inspire clinicians and neurogenetic researchers in the study and treatment of cerebellar disorders, by providing a resource for gathering background information and creating hypotheses. This database will prove to be useful in identifying genes with mutations associated with disorders which have yet to be modelled in mice, as demonstrated by the comparisons conducted in this study. One example is EBF3/Ebf3, which is a transcription factor belonging to the early B cell factor protein family involved in neuronal differentiation and maturation [31–33]. Deficits in EBF3 function result in hypotonia, ataxia and delayed development syndrome (HADDs) [34–37]. The core phenotypes include cerebellar ataxia, severe intellectual disability, subtle facial dysmorphism, strabismus, and vesicoureteric reflux, suggesting that EBF3 has a widespread developmental role. Additionally, EBF3 mutations have also been found to result in abnormal cerebellar foliation [38]. Mutations in EBF3 associated with HADDs has were found to occur in the DNA binding domain, resulting in reduced genome-wide binding and reduction of reporter gene expression in transactivation assays [35]. Ebf3 has previously been shown to dimerize with its paralog Ebf2 during development which has previously been associated with Purkinje cell migration and cerebellar patterning [39, 40]. However, the molecular processes regulated specifically by EBF3 during cerebellar development still remain elusive. A more in-depth examination of EBF3 function in the developing mouse cerebellum may help identify the mechanisms underlying HADDs pathogenesis as a result of EBF3 mutations and may lead to novel avenues for therapeutic intervention.

We believe that the Cerebellar Gene Database will reach its full potential with contributions from fellow researchers in the field. We encourage inputs, such as genes with novel functions in the context of the cerebellum and cerebellar phenotypes/disorders associated with genes in the existing database. The online resource will also have the capabilities to curate through directed searches and export custom datasets to help facilitate future research. These capabilities are outlined in our supplementary User Guide. Overall, our hope is that this becomes a living database that remains up to date with the advances in the genetic basis of cerebellar development and dysfunction.

## Supplementary Information

Below is the link to the electronic supplementary material.

Supplementary file1 (PDF 0.98 MB)

Supplementary file2 (XLSX 64 KB)

Supplementary file3 (PDF 121 KB)

## Data Availability

Data can be directly downloaded from the online database resource upon registration: https://cbgrits.org/Database/CerebellarGene

## References

[1] Sidman RL, Green MC, Appel SH (1965). Catalog of the neurological mutants of the mouse.

[2] McKusick VA (2007). Mendelian Inheritance in Man and its online version. OMIM Am J Human Gene.

[3] Diez-Roux G, Banfi S, Sultan M, Geffers L, Anand S, Rozado D, Magen A, Canidio E, Pagani M, Peluso I, Lin-Marq N, Koch M, Bilio M, Cantiello I, Verde R, De Masi C, Bianchi SA, Cicchini J, Perroud E, Ballabio A (2011). A high-resolution anatomical atlas of the transcriptome in the mouse embryo. PLoS Biol.

[4] Gong S, Zheng C, Doughty ML, Losos K, Didkovsky N, Schambra UB, Nowak NJ, Joyner A, Leblanc G, Hatten ME, Heintz N (2003). A gene expression atlas of the central nervous system based on bacterial artificial chromosomes. Nature.

[5] Ha T, Swanson D, Larouche M, Glenn R, Weeden D, Zhang P, Hamre K, Langston M, Phillips C, Song M, Ouyang Z, Chesler E, Duvvurru S, Yordanova R, Cui Y, Campbell K, Ricker G, Phillips C, Homayouni R, Goldowitz D (2015). CbGRiTS: cerebellar gene regulation in time and space. Dev Biol.

[6] Sato A, Sekine Y, Saruta C, Nishibe H, Morita N, Sato Y, Sadakata T, Shinoda Y, Kojima T, Furuichi T (2008). Cerebellar development transcriptome database (CDT-DB): profiling of spatio-temporal gene expression during the postnatal development of mouse cerebellum. Neural Networks: Off J Int Neural Network Soc.

[7] Sunkin SM, Ng L, Lau C, Dolbeare T, Gilbert TL, Thompson CL, Hawrylycz M, Dang C. Allen Brain Atlas: an integrated spatio-temporal portal for exploring the central nervous system. Nucleic Acids Res. (2013);41(Database issue), D996-D1008. 10.1093/nar/gks1042. 10.1093/nar/gks1042 PMC3531093 23193282

[8] Aldinger KA, Thomson Z, Phelps IG, Haldipur P, Deng M, Timms AE, Hirano M, Santpere G, Roco C, Rosenberg AB, Lorente-Galdos B, Gulden FO, O’Day D, Overman LM, Lisgo SN, Alexandre P, Sestan N, Doherty D, Dobyns WB, Millen KJ (2021). Spatial and cell type transcriptional landscape of human cerebellar development. Nat Neurosci.

[9] Carter RA, Bihannic L, Rosencrance C, Hadley JL, Tong Y, Phoenix TN, Natarajan S, Easton J, Northcott PA, Gawad C (2018). A single-cell transcriptional atlas of the developing murine cerebellum. Current Biology: CB.

[10] Ha TJ, Zhang PGY, Robert R, Yeung J, Swanson DJ, Mathelier A, Wasserman WW, Im S, Itoh M, Kawaji H, Lassmann T, Daub CO, Arner E, Carninci P, Hayashizaki Y, Forrest ARR, Goldowitz D (2019). Identification of novel cerebellar developmental transcriptional regulators with motif activity analysis. BMC Genomics.

[11] Khouri-Farah N, Guo Q, Morgan K, Shin J, Li JYH. Integrated single-cell transcriptomic and epigenetic study of cell state transition and lineage commitment in embryonic mouse cerebellum. Sci Adv. (2022);8(13), eabl9156. 10.1126/sciadv.abl9156. 10.1126/sciadv.abl9156 PMC10938588 35363520

[12] Vladoiu MC, El-Hamamy I, Donovan LK, Farooq H, Holgado BL, Sundaravadanam Y, Ramaswamy V, Hendrikse LD, Kumar S, Mack SC, Lee JJY, Fong V, Juraschka K, Przelicki D, Michealraj A, Skowron P, Luu B, Suzuki H, Morrissy AS, Taylor MD (2019). Childhood cerebellar tumours mirror conserved fetal transcriptional programs. Nature.

[13] Grinberg I, Northrup H, Ardinger H, Prasad C, Dobyns WB, Millen KJ (2004). Heterozygous deletion of the linked genes ZIC1 and ZIC4 is involved in Dandy-Walker malformation. Nat Genet.

[14] Hamilton BA, Frankel WN, Kerrebrock AW, Hawkins TL, FitzHugh W, Kusumi K, Russell LB, Mueller KL, van Berkel V, Birren BW, Kruglyak L, Lander ES (1996). Disruption of the nuclear hormone receptor RORalpha in staggerer mice. Nature.

[15] Hamre KM, Goldowitz D (1997). meander tail acts intrinsic to granule cell precursors to disrupt cerebellar development: analysis of meander tail chimeric mice. Development (Cambridge, England).

[16] Yeung J, Ha TJ, Swanson DJ, Goldowitz D (2016). A novel and multivalent role of Pax6 in cerebellar development. J Neurosci: Off J Soc Neurosci.

[17] Badura A, Verpeut JL, Metzger JW, Pereira TD, Pisano TJ, Deverett B, Bakshinskaya DE, Wang SS. Normal cognitive and social development require posterior cerebellar activity. eLife. (2018);7 10.7554/eLife.36401. 10.7554/eLife.36401 PMC6195348 30226467

[18] Broussard GJ, Kislin M, Jung C, Wang SS. A flexible platform for monitoring cerebellum-dependent sensory associative learning. J Visualized Exp JoVE. (2022);(179) 10.3791/63205. 10.3791/63205 PMC9118201 35129170

[19] Ernst TM, Brol AE, Gratz M, Ritter C, Bingel U, Schlamann M, Maderwald S, Quick HH, Merz CJ, Timmann D. The cerebellum is involved in processing of predictions and prediction errors in a fear conditioning paradigm. eLife. (2019);8 10.7554/eLife.46831. 10.7554/eLife.46831 PMC6715348 31464686

[20] Kelly E, Meng F, Fujita H, Morgado F, Kazemi Y, Rice LC, Ren C, Escamilla CO, Gibson JM, Sajadi S, Pendry RJ, Tan T, Ellegood J, Basson MA, Blakely RD, Dindot SV, Golzio C, Hahn MK, Katsanis N, Tsai PT (2020). Regulation of autism-relevant behaviors by cerebellar-prefrontal cortical circuits. Nat Neurosci.

[21] Low AYT, Goldstein N, Gaunt JR, Huang K, Zainolabidin N, Yip AKK, Carty JRE, Choi JY, Miller AM, Ho HST, Lenherr C, Baltar N, Azim E, Sessions OM, Ch’ng TH, Bruce AS, Martin LE, Halko MA, Brady RO, Betley JN (2021). Reverse-translational identification of a cerebellar satiation network. Nature.

[22] Schmahmann JD (2019). The cerebellum and cognition. Neurosci Lett.

[23] Strick PL, Dum RP, Fiez JA (2009). Cerebellum and nonmotor function. Annu Rev Neurosci.

[24] Van Overwalle F, Manto M, Cattaneo Z, Clausi S, Ferrari C, Gabrieli JDE, Guell X, Heleven E, Lupo M, Ma Q, Michelutti M, Olivito G, Pu M, Rice LC, Schmahmann JD, Siciliano L, Sokolov AA, Stoodley CJ, van Dun K, Leggio M (2020). Consensus paper: cerebellum and social cognition. Cerebellum (London, England).

[25] Leto K, Arancillo M, Becker EBE, Buffo A, Chiang C, Ding B, Dobyns WB, Dusart I, Haldipur P, Hatten ME, Hoshino M, Joyner AL, Kano M, Kilpatrick DL, Koibuchi N, Marino S, Martinez S, Millen KJ, Millner TO, Miyata T, Parmigiani E, Schilling K, Sekerková G, Sillitoe RV, Sotelo C, Uesaka N, Wefers A, Wingate RJT, Hawkes R (2016). Consensus Paper: Cerebellar Development. The Cerebellum.

[26] Koziol LF, Budding D, Andreasen N, D’Arrigo S, Bulgheroni S, Imamizu H, Ito M, Manto M, Marvel C, Parker K, Pezzulo G, Ramnani N, Riva D, Schmahmann J, Vandervert L, Yamazaki T (2014). Consensus paper: the cerebellum’s role in movement and cognition. Cerebellum (London, England).

[27] Aruga J, Yokota N, Hashimoto M, Furuichi T, Fukuda M, Mikoshiba K (1994). A novel zinc finger protein, zic, is involved in neurogenesis, especially in the cell lineage of cerebellar granule cells. J Neurochem.

[28] Jeste SS, Sahin M, Bolton P, Ploubidis GB, Humphrey A (2008). Characterization of autism in young children with tuberous sclerosis complex. J Child Neurol.

[29] Reith RM, McKenna J, Wu H, Hashmi SS, Cho S, Dash PK, Gambello MJ (2013). Loss of Tsc2 in Purkinje cells is associated with autistic-like behavior in a mouse model of tuberous sclerosis complex. Neurobiol Dis.

[30] Tsai PT, Hull C, Chu Y, Greene-Colozzi E, Sadowski AR, Leech JM, Steinberg J, Crawley JN, Regehr WG, Sahin M (2012). Autistic-like behaviour and cerebellar dysfunction in Purkinje cell Tsc1 mutant mice. Nature.

[31] Chiara F, Badaloni A, Croci L, Yeh ML, Cariboni A, Hoerder-Suabedissen A, Consalez GG, Eickholt B, Shimogori T, Parnavelas JG, Rakić S (2012). Early B-cell factors 2 and 3 (EBF2/3) regulate early migration of Cajal-Retzius cells from the cortical hem. Dev Biol.

[32] Chowdhury TG, Jimenez JC, Bomar JM, Cruz-Martin A, Cantle JP, Portera-Cailliau C (2010). Fate of cajal-retzius neurons in the postnatal mouse neocortex. Front Neuroanat.

[33] Liberg D, Sigvardsson M, Akerblad P (2002). The EBF/Olf/Collier family of transcription factors: regulators of differentiation in cells originating from all three embryonal germ layers. Mol Cell Biol.

[34] Chao H, Davids M, Burke E, Pappas JG, Rosenfeld JA, McCarty AJ, Davis T, Wolfe L, Toro C, Tifft C, Xia F, Stong N, Johnson TK, Warr CG, Yamamoto S, Adams DR, Markello TC, Gahl WA, Bellen HJ, Malicdan MCV (2017). A syndromic neurodevelopmental disorder caused by de novo variants in EBF3. Am J Hum Genet.

[35] Harms FL, Girisha KM, Hardigan AA, Kortüm F, Shukla A, Alawi M, Dalal A, Brady L, Tarnopolsky M, Bird LM, Ceulemans S, Bebin M, Bowling KM, Hiatt SM, Lose EJ, Primiano M, Chung WK, Juusola J, Akdemir ZC, Kutsche K (2017). Mutations in EBF3 disturb transcriptional profiles and cause intellectual disability, ataxia, and facial dysmorphism. Am J Hum Genet.

[36] Sleven H, Welsh SJ, Yu J, Churchill MEA, Wright CF, Henderson A, Horvath R, Rankin J, Vogt J, Magee A, McConnell V, Green A, King MD, Cox H, Armstrong L, Lehman A, Nelson TN, Williams J, Clouston P, Németh AH (2017). De novo mutations in EBF3 cause a neurodevelopmental syndrome. Am J Hum Genet.

[37] Tanaka AJ, Cho MT, Willaert R, Retterer K, Zarate YA, Bosanko K, Stefans V, Oishi K, Williamson A, Wilson GN, Basinger A, Barbaro-Dieber T, Ortega L, Sorrentino S, Gabriel MK, Anderson IJ, Sacoto MJG, Schnur RE, Chung WK. De novo variants in EBF3 are associated with hypotonia, developmental delay, intellectual disability, and autism. Cold Spring Harbor Mol Case Stud. (2017);3(6) 10.1101/mcs.a002097. 10.1101/mcs.a002097 PMC5701309 29162653

[38] D’Arrigo S, Moscatelli M, Ciaccio C, Pantaleoni C, Castello R, Chiapparini L (2020). Abnormal cerebellar foliation in EBF3 mutation. Neurology.

[39] Badaloni A, Casoni F, Croci L, Chiara F, Bizzoca A, Gennarini G, Cremona O, Hawkes R, Consalez GG (2019). Dynamic expression and new functions of early B cell factor 2 in cerebellar development. Cerebellum (London, England).

[40] Croci L, Chung S, Masserdotti G, Gianola S, Bizzoca A, Gennarini G, Corradi A, Rossi F, Hawkes R, Consalez GG (2006). A key role for the HLH transcription factor EBF2COE2, O/E-3 in Purkinje neuron migration and cerebellar cortical topography. Development (Cambridge, England).

