# Supplementary material for: The Cerebellar Gene Database: a Collective Database of Genes Critical for Cerebellar Development
Source: Cerebellum. 2022 Jul 20;21(4):606–14. doi: 10.1007/s12311-022-01445-w (PMC9325837; doi:10.1007/s12311-022-01445-w)
Supplement: Supplementary file 1 — (PDF 0.98 MB) [file 12311_2022_1445_MOESM1_ESM.pdf]

CEREBELLAR GENE DATABASE USER GUIDE

1. PREFACE

1.1 Online Database Access

The Cerebellar Gene Database can be accessed online at:
https://cbgrits.org/Database/CerebellarGene

1.2 Description of the Cerebellar Gene Database

The Cerebellar Gene Database compilation of mouse and human genes critical for the development and function of the cerebellum. These genes were curated based on an observed cerebellar phenotype as a result of genetic perturbation, either experimentally (mouse) or through inheritance (human). There are three sections of the Cerebellar Gene Database: 1) Mouse Cerebellar Genes, 2) Mouse Cerebellar Phenotypes and 3) Human Cerebellar Genes. The datasets from each section can be queried, filtered and exported for future hypothesis formation and dataset association.

If you are using this database, please consult and cite **Ramirez et al., The Cerebellar Gene Database, Cerebellum, 2022**

2. BASICS OF USE

2.1 Interface

The home page provides several useful links to register for an account, accessing the contribution page and contacting the authors (**Figure 1**). The Cerebellar Gene Database consists of three sections: Mouse Cerebellar Genes, Mouse Cerebellar Phenotypes and Human Cerebellar Genes. The contents of these sections are detailed in our publication in Tables 1, 2 and 3, respectively. These different sections can be accessed by clicking on the respective tabs (**Figure 1**).

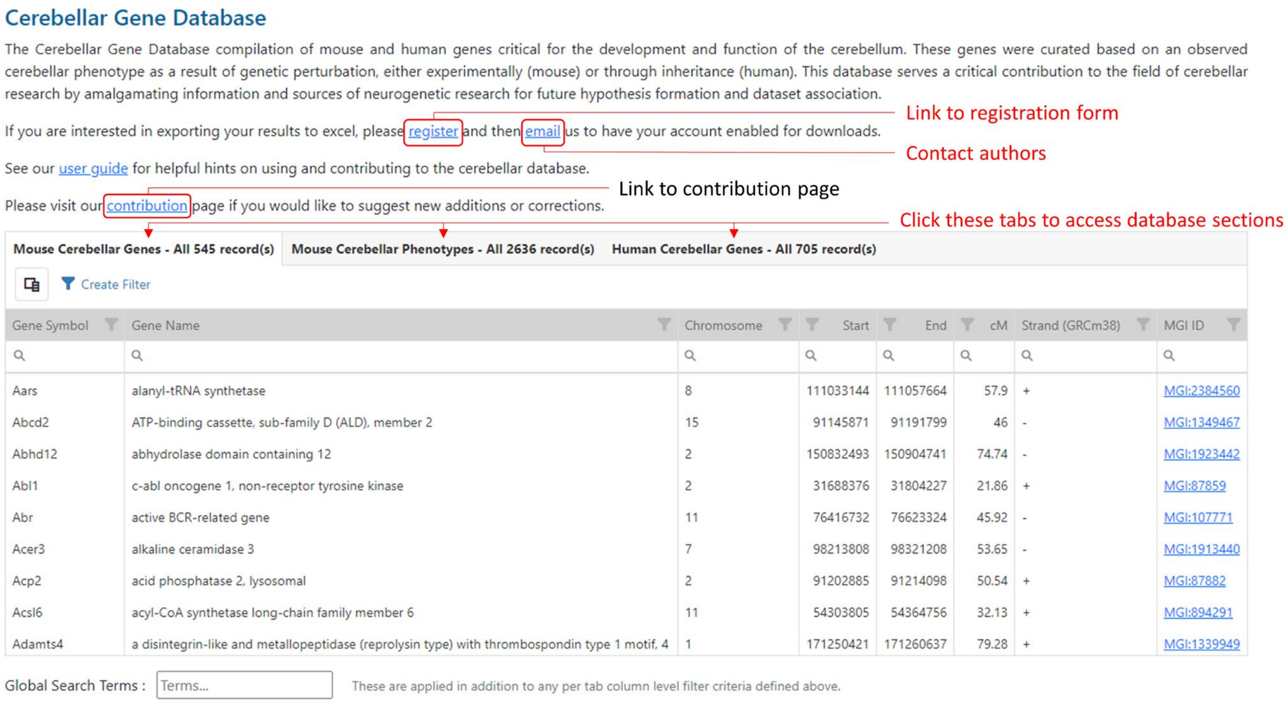

Figure 1. The Cerebellar Gene Database interface. Red boxes highlight links for account registration, contacting the authors and the contribution page. Red arrows indicate the tabs used to access the different sections of the database.

2.2 Cerebellar Gene Database Sections

1) Mouse Cerebellar Genes: Contains basic information about the mouse genes that when perturbed result in a cerebellar phenotype. Description of the columns can be found in Table 1 below:

Column Name	Description
**Gene Symbol**	Gene symbol as appears on MGI
**Gene Name**	Name of the gene as appears on MGI
**Chromosome**	Chromosome in which the gene is located

**Start**	Starting position of the gene in the corresponding chromosome
**End**	Ending position of the gene in the corresponding chromosome
**cM**	Gene in centimorgans
**Strand (GRCm38)**	Strand orientation of the gene (exists as “+” or “-”)
**MGI ID**	Unique ID number given to each gene by MGI (*http://www.informatics.jax.org/marker/MGI*)

Table 1. Overview of the contents of the “Mouse Cerebellar Genes” section of the Cerebellar Gene Database

2) Mouse Cerebellar Phenotypes: Contains cerebellar phenotypes identified after perturbation of gene function, corresponding links to the MGI database and links to sources. Description of the columns can be found below in Table 2:

Column Name	Description
**Gene Symbol**	Gene symbol (as appears on MGI)
**Gene Name**	Name of the gene (as appears on MGI)
**MGI ID**	Unique ID number given to each gene by MGI
**Phenotype**	Phenotype implicated in gene’s loss of function (e.g., knockout), unless specified otherwise
**Mouse Phenotype**	Unique ID number given to each phenotype by MGI
**Source**	ID number (from MGI or PubMed) of study/publication detailing the phenotype observed in the gene’s loss of function

Table 2. Overview of the contents of the “Mouse Cerebellar Phenotypes" section of the Cerebellar Gene Database.

3) Human Cerebellar Genes: Contains human genes associated with disorders with cerebellar phenotypes. This section contains general information on the genes, description of the disorders, links to the OMIM database and mouse orthologs. Description of the columns can be found below in Table 3:

Gene Symbol / Locus	Gene or Locus symbol as appears on OMIM
**Gene / Locus Name**	Name of the gene and/or locus associated with the phenotype
**Gene / Locus MIM Number**	ID used to search the gene/locus directly on OMIM
**Cytogenetic Location**	Cytogenetic location of the gene associated with the phenotype
**Genomic Coordinates (NCBI/GRCh38)**	Location of the gene, denoted in the format of [Chromosome]:[Starting position]-[Ending position]
**Disorder / Phenotype**	Name of disorder or phenotype as can be found on OMIM

**Phenotype MIM Number**	ID number of the disorder/phenotype on OMIM
**Inheritance**	Inheritance pattern of the disease/phenotype
**Phenotype Map Key**	A number from 1–4 stands for the following: “(1) the disorder was positioned by mapping of the wild-type gene; (2) the disease phenotype itself was mapped; (3) the molecular basis of the disorder is known; (4) the disorder is a chromosome deletion or duplication syndrome,” retrieved from *https://www.omim.org/help/faq*.
**Comments**	Comments/additional information regarding the phenotype
**Mouse Gene (from MGI)**	Mouse homolog (as can be found on MGI) of the gene responsible for the human phenotype

Table 3. Overview of the contents of the “Human Cerebellar Phenotypes” section of the Cerebellar Gene Database.

2.3 External Links

In each section of the database, unique MGI IDs and OMIM IDs are hyperlinked. Clicking on these links will redirect the user to either the MGI or OMIM websites.

The ‘**MGI ID**’ column contains links that will direct you to the ‘Gene Detail’ webpage for the corresponding gene in that row. The Gene Detail webpage provides a comprehensive overview of gene location, function, expression, protein information, mutations/alleles and their associated phenotypes and references.

The ‘**Mouse Phenotype ID**’ column contains links that will direct you to the ‘Phenotype Term Detail’ webpage for the corresponding phenotype in that row. This page is part of the MGI ‘Mammalian Phenotype Browser’ and provides a detailed definition of a given phenotype, a link to other genotypes/annotations that result in that phenotype and other closely related phenotypes.

The ‘**Gene/Locus MIM Number**’ column contains links that will direct you to the OMIM entry page for the corresponding gene/locus in that row. This page will provide more information on the gene/locus, gene-phenotype relationships, allelic variants and corresponding references.

The ‘**Phenotype MIM Number**’ column contains links that will direct you to the OMIM entry page for the corresponding phenotype/disorder. This page will provide more information on the disorder such as clinical features, inheritance patterns, gene-phenotype relationships, and corresponding references.

3. REGISTERING AND ACCOUNT CREATION

In order to export datasets and contribute to the Cerebellar Gene Database, registration for an account is required. Registration can be completed by clicking on the register link and filling out the information in the online form.

4. EXPORTING DATASETS

To enable exporting of the datasets, click on the 'email' link on the database webpage or email info@cbgrits.org directly. Please provide the email address used for registration and indicate in the subject line 'Export Permission Request – Cerebellar Gene Database' (this should automatically be generated if the link was used). An email will be sent back to confirm that downloading datasets is enabled for the account.

After receiving this email and logging into the account, a new icon will appear at the top left of the data table (**Figure 1**). Clicking this icon will download the dataset. To download a curated dataset, apply the filters of interest and then click the export icon.

5. CONTRIBUTING TO THE CEREBELLAR GENE DATABASE

The Cerebellar Gene Database is meant to be an interactive and collaborative research effort in order to create a centralized resource for mouse and human genes with relevance to cerebellar development and function. We encourage input and contributions from the research community to any of the three sections of the database.

Inputs may include:

- Mouse and human genes/loci that when perturbed result in a cerebellar phenotype.
- Cerebellar phenotypes or human disorders with cerebellar phenotypes associated with genes already in the database or genes that have yet to be cataloged.

In order to enable suggesting new inputs to the database, an account must be created followed by email requesting that the account be enabled for contributions. After this is complete, the contribution page will show a table similar to the original database but with different icons in the top left side of the table (**Figure 2**).

To suggest new contributions to the Cerebellar Gene Database, click on the '+' sign to add a new row to the database. In the new row, enter the corresponding information about the gene/loci or phenotype/disorder as shown in the example in **Figure 2**.

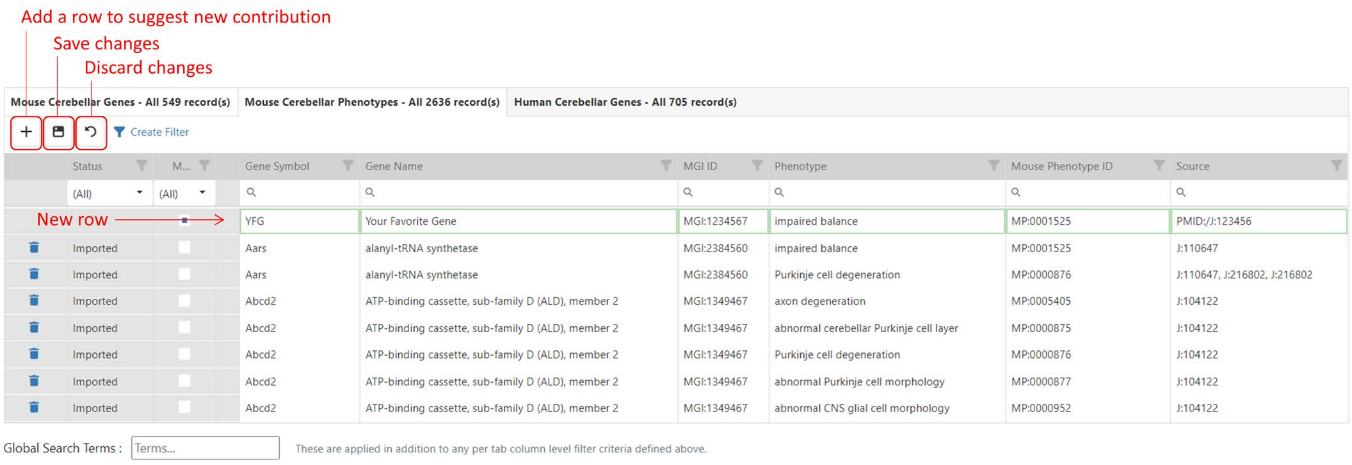

Figure 2. An example of an new entry into the database on the Contribution

To save this entry, click on the save changes icon.

To discard the entry, click on the discard changes icon.

Please email info@cbgrits.org once the submission is ready for review.

Note that contributions need to be administratively approved.

With any contribution, please provide detailed information for all columns in the corresponding format. Gene and phenotype information and nomenclature can typically be found on **MGI** or **OMIM** websites.

6. SEARCHING AND FILTERING

The sections of the Cerebellar Gene Database can be curate/filtered using either the Global Search box, Column Filters or Filter Builder functions.

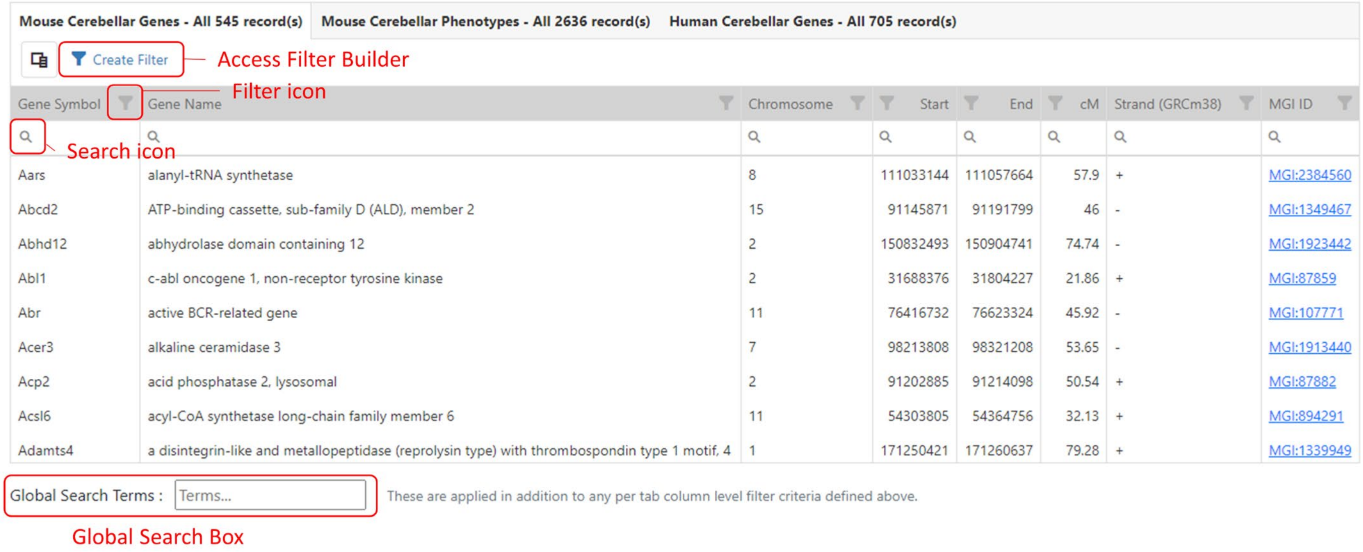

Gene Symbol	Gene Name	Chromosome	Start	End	cM	Strand (GRCm38)	MGI ID
Aars	alanyl-tRNA synthetase	8	111033144	111057664	57.9	+	MGI:2384560
Abcd2	ATP-binding cassette, sub-family D (ALD), member 2	15	91145871	91191799	46	-	MGI:1349467
Abhd12	abhydrolase domain containing 12	2	150832493	150904741	74.74	-	MGI:1923442
Abl1	c-abl oncogene 1, non-receptor tyrosine kinase	2	31688376	31804227	21.86	+	MGI:87859
Abr	active BCR-related gene	11	76416732	76623324	45.92	-	MGI:107771
Acer3	alkaline ceramidase 3	7	98213808	98321208	53.65	-	MGI:1913440
Acp2	acid phosphatase 2, lysosomal	2	91202885	91214098	50.54	+	MGI:87882
Acsl6	acyl-CoA synthetase long-chain family member 6	11	54303805	54364756	32.13	+	MGI:894291
Adamts4	a disintegrin-like and metallopeptidase (reprolysin type) with thrombospondin type 1 motif, 4	1	171250421	171260637	79.28	+	MGI:1339949

Figure 3. Searching and filtering options for the Cerebellar Gene Database. Red boxes highlight the various search and filter options.

3.1 Global Search

The Global Search bar will filter the three sections of the Cerebellar Gene Database simultaneously. Each dataset will be filtered for any row containing the text entered into the search box. This is a useful tool for filtering the entire database for genes or disorders of interest.

The Global Search is located at the bottom of the page (**Figure 3**).

As noted on the page, the Global Search is applied in addition to any per tab column filter criteria, as described below.

3.2 Column Filters

Each section of the Cerebellar Gene Database can be filtered by criteria applied to individual columns. At the top of each column, there is a **filter icon** adjacent to the column name and a text box with a **search icon** (**Figure 3**).

Clicking on the **filter icon** will open a **pop-up box** containing all entries in that column. Checking the box beside entries of interest will filter the dataset for rows containing those exact values.

The textbox at the top of each column can also be used to filter the dataset based on entries in that column. As the default setting, using this textbox will search the column for any entries that '**contain'** the queried text.

The parameters of the column-based filtering can be changed by clicking on the search icon. This will open a drop down menu containing six types of filters:

- **Contains:** Filters for any entries containing searched text.
- **Does not contain**: Filters for entries that do not contain searched text.
- **Starts with:** Filters for entries that start with searched text.
- **Ends with:** Filters for entries that end with searched text.
- **Equals**: Filters for entries that exactly match the searched text.
- **Does not equal:** Filters for entries that do not exactly match the searched text.

Column-based criteria can be applied to multiple columns simultaneously, further refining the dataset.

As the default setting, filtering using multiple columns will search the dataset for any rows that fulfill **all** the search criteria applied. A summary of your search criteria will be displayed above the table (**Figure 4**).

An example of this can be found in **Figure 4**. This screenshot displays the 'Mouse Cerebellar Phenotypes' table, with the 'Gene Symbol' column filtered for any text starting with 'cacna' and the 'Phenotype' column filtered for any entries containing 'purkinje'. Applying these filters identified rows that satisfy both these criteria, as indicated by the search summary.

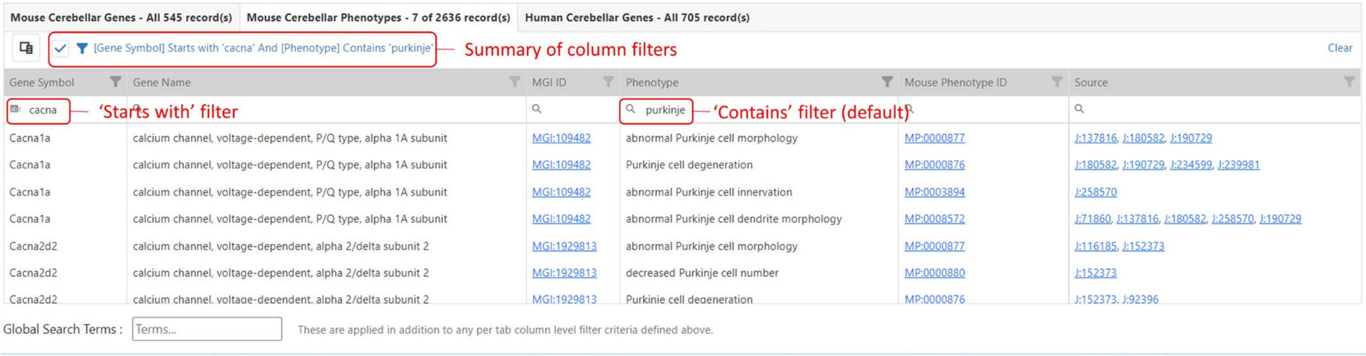

Figure 4. Example of using the Column Filters to curate the Cerebellar Gene Database. The red boxes highlight the search summary and type of filters used to search each column of interest.

3.3 Filter Builder (single condition, multiple condition, nested conditions search)

The Filter Builder can be utilized to fully customize the search criteria. This may be important for users searching for a specific set of phenotypes or genes of interest. This can be opened by clicking on the 'Create Filter' link displayed above the table (**Figure 3**).

Interface. The Filter Builder will initially display three things: the 'Expression' description, a green '+' sign and the group modifier in a red box (**Figure 5**).

The 'Expression' description will display a summary of your filter criteria.

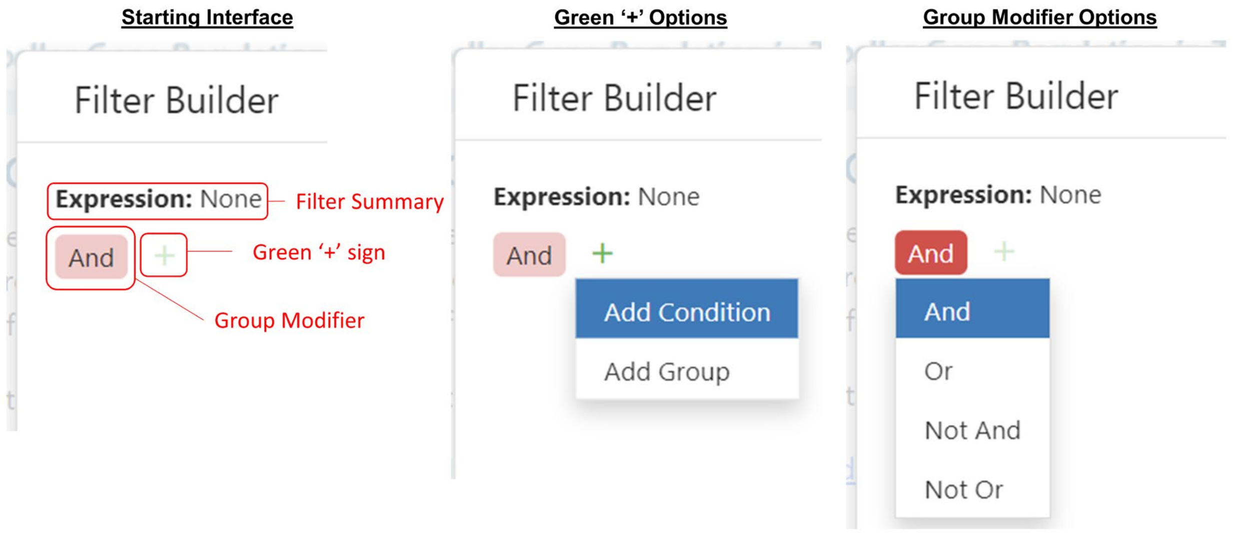

Figure 5. Filter Builder starting options. **Left**: The initial interface of the Filter Builder. The red boxes highlight the filter summary, and the icons used to access the filter options. **Middle**: The options that are displayed after clicking on the green '+'. **Right**: The options that are displayed after clicking on the group modifier box.

In order to fully display the capabilities of the Filter Builder, three examples are outlined below: Single Condition, Multiple Condition and Nested Conditions searches.

3.3.1 Single Condition Search

To get started, clicking on the green '+' sign will open up a drop down menu with two options: Add Condition and Add Group (**Figure 5**). This example will utilize the Add Condition option.

Add Condition allows the user to set a search criterion for a column in the current section of the Cerebellar Database within this search group.

- The column can be selected by clicking on the blue box which opens a drop down menu.
- The criteria modifier can be selected by clicking the green box which opens a drop down menu with the same options as the column-based filters.
- The search text can be typed into the grey textbox.

Figure 6 displays an example of a basic single condition search in the 'Mouse Cerebellar Phenotypes' section of the database. This search is filtering the '**Phenotype**' column for any row that '**Contains**' the word '**granule**'.

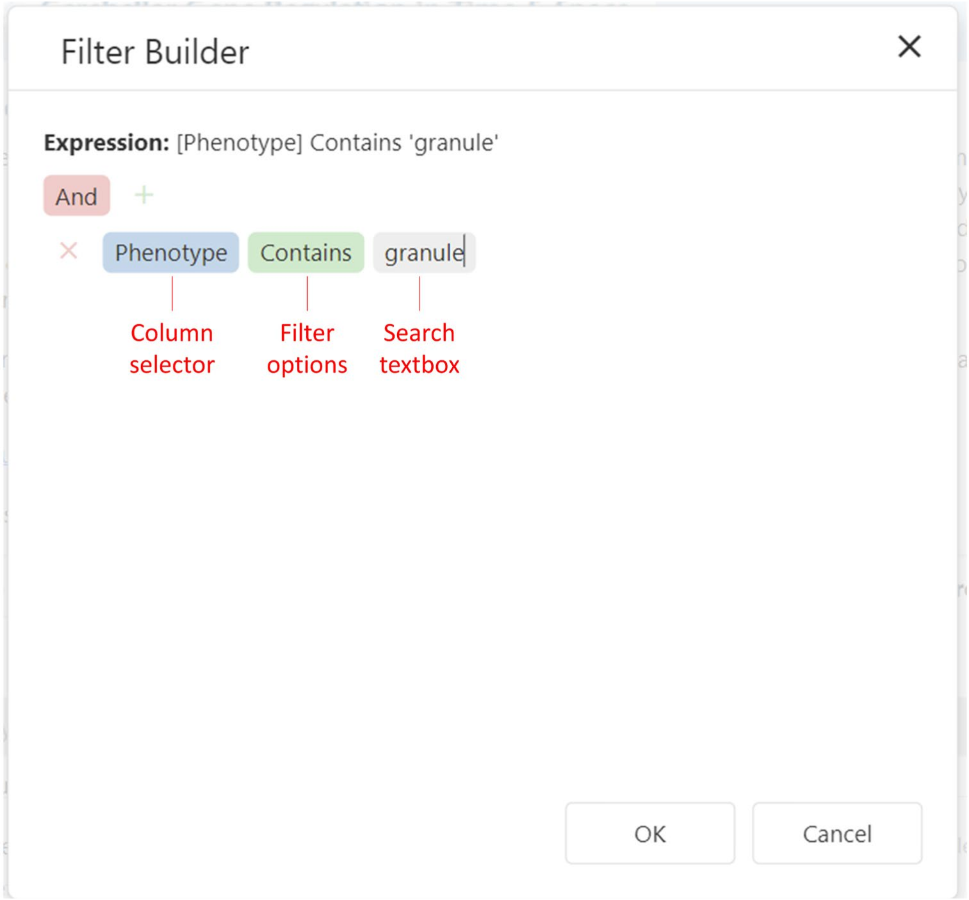

Figure 6. Example of a Single Condition Search in the Filter Builder. Red boxes highlight the different options that can be used to customize a search using the Filter Builder.

3.3.2 Multiple Conditions Search

Multiple search conditions can be added which will further refine the dataset. Clicking the green '+' sign and clicking Add Condition will allow multiple search criteria to be applied to the dataset. The added criteria can be applied to either the same column or to multiple different columns.

These conditions are currently within the same 'search group'.

By default, the 'group modifier' is set to 'And', which will result in a search that identifies rows that fulfill all the search conditions. The modifier can be changed by clicking the red search group modifier box which will open a drop down menu with the following options:

- **'And':** Filter for rows fulfilling all search conditions.
- **'Or':** Filter for rows containing any of the search conditions separately.
- **'Not And':** Filter for rows that DO NOT fulfill **all** search conditions
- **'Not Or':** Filter for rows that DO NOT fulfill any of the search conditions separately

Figure 7 displays an example of a multiple conditions search in the 'Mouse Cerebellar Phenotypes' section of the database. Building off the previous example, a second search condition is added, filtering the 'Phenotype' column for granule and the 'Gene Symbol' column for 'neurod'.

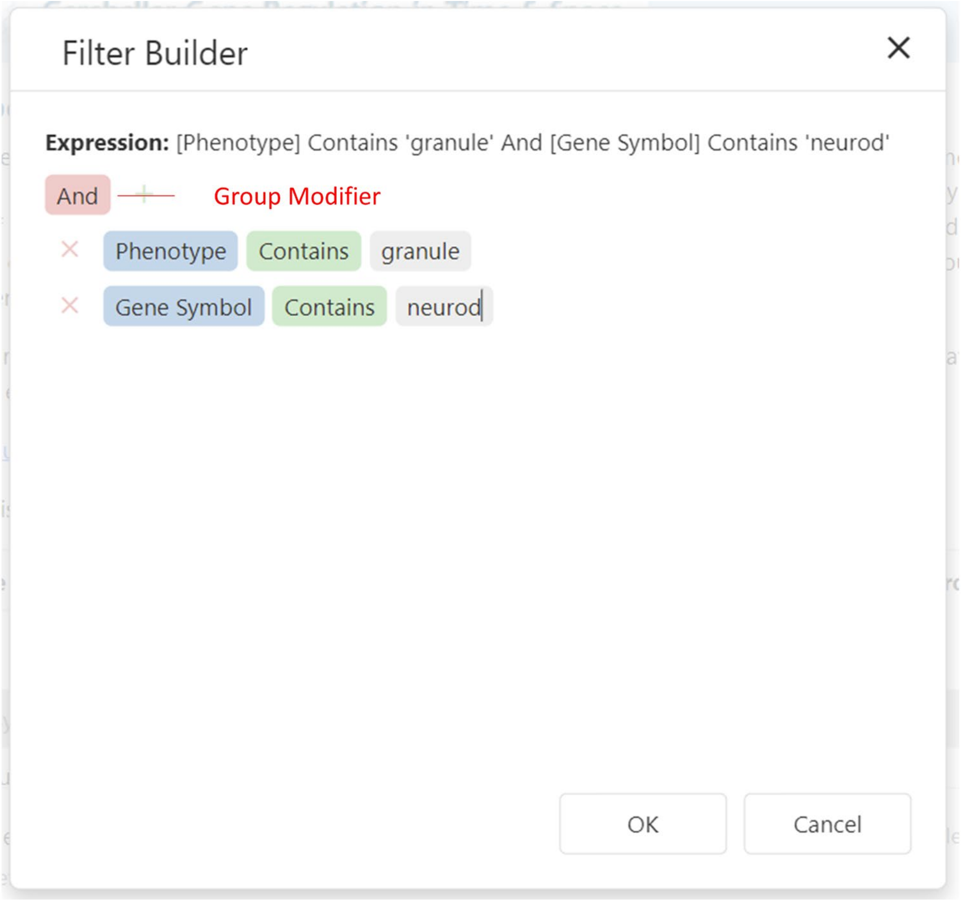

Figure 7. Example of a Multiple Conditions Search. Red box is highlighting the Group Modifier which can be used to change the parameters of the search.

3.3.3 Nested Conditions Search

The filtering criteria for multiple conditions can be further customized through a nested conditions search.

This type of search is conducted by creating multiple ‘filter groups’.

Creating these groups can be achieved by clicking the green ‘+’ sign and clicking ‘**Add Group**’.

A new group addition is signified by the appearance of a new red filter modifier box and green ‘+’ sign, just under the original set (**Figure 8**).

Multiple groups can be added by using the original top-most green ‘+’.

Conditions can be added into each of the groups by clicking on the groups respective green ‘+’, and clicking Add Condition.

The nested search can be modified by adjusting the top-most group modifier. This will modify the filter for the multiple search groups.

Figure 8 shows an example of a nested conditions search utilizing multiple groups and multiple conditions for the ‘Mouse Cerebellar Phenotypes’. In this example, two groups were created. In the first group, the ‘Phenotype’ column will be filtered for any entries containing ‘granule and ‘balance’. In the second group, the ‘Gene Symbol’ column will be filtered for any entries containing ‘neurod or ‘pax. Combined this search will identify any rows satisfying two sets of criteria for two separate columns.

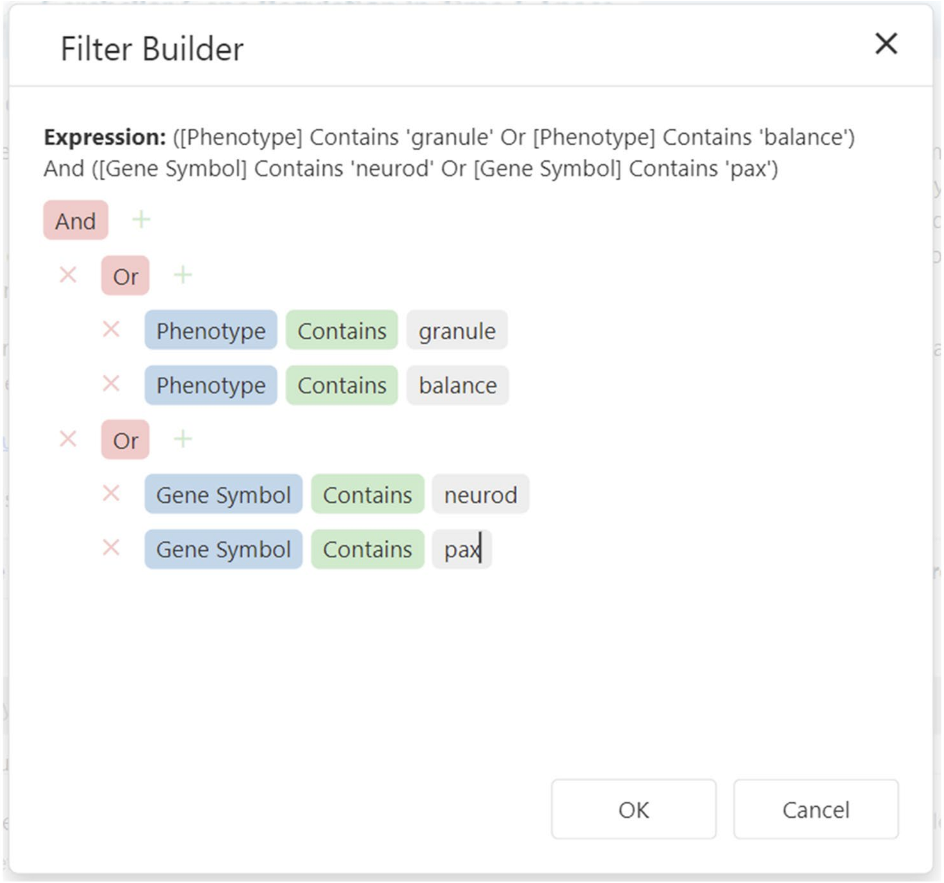

Figure 8 Example of a Nested Conditions Search.
