# Supplementary material for: The Cerebellar Gene Database: a Collective Database of Genes Critical for Cerebellar Development
Source: Cerebellum. 2022 Jul 20;21(4):606–14. doi: 10.1007/s12311-022-01445-w (PMC9325837; doi:10.1007/s12311-022-01445-w)
Supplement: Supplementary file 3 — (PDF 121 KB) [file 12311_2022_1445_MOESM3_ESM.pdf]

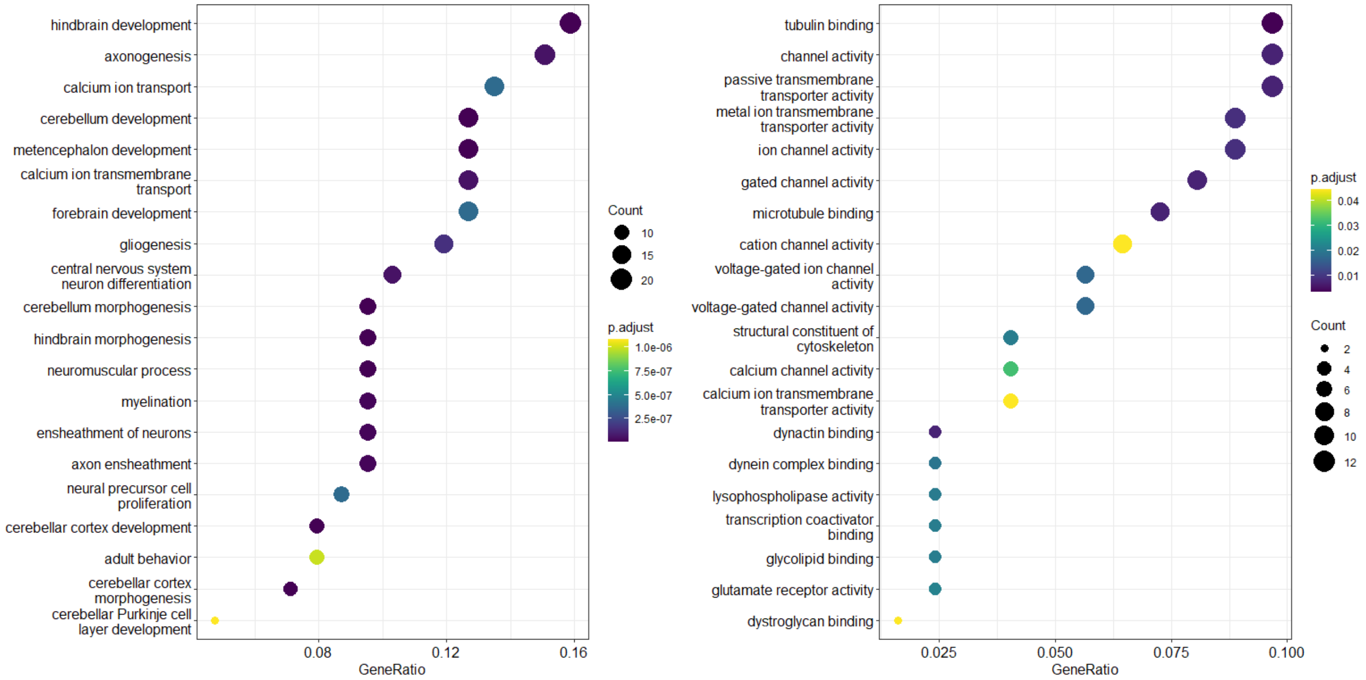

Supplemental Figure 1. Dot plots representing the enriched Biological Function (left) and Molecular Processes (right) Gene Ontology terms for the 126 genes overlapping between the mouse and human cerebellar gene lists. The x-axis (GeneRatio) represents the genes of interest in the gene GO category divided by the total number genes of interest. The color scale represents adjusted p-value of the enrichment of each GO term in the gene list. Dot size (Count) represents the number of genes from the gene list in the GO category.
